# Modeling multifunctionality of genes with secondary gene co-expression networks in human brain provides novel disease insights

**DOI:** 10.1093/bioinformatics/btab175

**Published:** 2021-03-18

**Authors:** Juan A Sánchez, Ana L Gil-Martinez, Alejandro Cisterna, Sonia García-Ruíz, Alicia Gómez-Pascual, Regina H Reynolds, Mike Nalls, John Hardy, Mina Ryten, Juan A Botía

**Affiliations:** Departamento de Ingeniería de la Información y las Comunicaciones, Universidad de Murcia, Murcia E-30100, Spain; Department of Neurodegenerative Diseases, UCL Institute of Neurology, London WC1E 6BT, UK; Departamento de Ingeniería de la Información y las Comunicaciones, Universidad de Murcia, Murcia E-30100, Spain; Department of Neurodegenerative Diseases, UCL Institute of Neurology, London WC1E 6BT, UK; Departamento de Ingeniería de la Información y las Comunicaciones, Universidad de Murcia, Murcia E-30100, Spain; Department of Neurodegenerative Diseases, UCL Institute of Neurology, London WC1E 6BT, UK; Laboratory of Neurogenetics, Molecular Genetics Section, Laboratory of Neurogenetics, National Institute on Aging, National Institutes of Health, Bethesda, MD 20892, USA; Data Tecnica International, Glen Echo, MD 20812, USA; Department of Neurodegenerative Diseases, UCL Institute of Neurology, London WC1E 6BT, UK; Department of Neurodegenerative Diseases, UCL Institute of Neurology, London WC1E 6BT, UK; Departamento de Ingeniería de la Información y las Comunicaciones, Universidad de Murcia, Murcia E-30100, Spain; Department of Neurodegenerative Diseases, UCL Institute of Neurology, London WC1E 6BT, UK

## Abstract

**Motivation:**

Co-expression networks are a powerful gene expression analysis method to study how genes co-express together in clusters with functional coherence that usually resemble specific cell type behavior for the genes involved. They can be applied to bulk-tissue gene expression profiling and assign function, and usually cell type specificity, to a high percentage of the gene pool used to construct the network. One of the limitations of this method is that each gene is predicted to play a role in a specific set of coherent functions in a single cell type (i.e. at most we get a single <gene, function, cell type> for each gene). We present here GMSCA (Gene Multifunctionality Secondary Co-expression Analysis), a software tool that exploits the co-expression paradigm to increase the number of functions and cell types ascribed to a gene in bulk-tissue co-expression networks.

**Results:**

We applied GMSCA to 27 co-expression networks derived from bulk-tissue gene expression profiling of a variety of brain tissues. Neurons and glial cells (microglia, astrocytes and oligodendrocytes) were considered the main cell types. Applying this approach, we increase the overall number of predicted triplets <gene, function, cell type> by 46.73%. Moreover, GMSCA predicts that the SNCA gene, traditionally associated to work mainly in neurons, also plays a relevant function in oligodendrocytes.

**Availabilityand implementation:**

The tool is available at GitHub, https://github.com/drlaguna/GMSCA as open-source software.

**Supplementary information:**

Supplementary data are available at *Bioinformatics* online.

## 1 Introduction

Gene co-expression networks (GCN) are a combination of gene clusters and graph networks, based on the correlation of mRNA levels from gene expression profiling (Botía et al., 2017; Langfelder and Horvath, 2008; Miller et al., 2010; Oldham et al., 2008). Genes appearing together in a cluster or as neighbors in the network are said to be co-expressed. GCN analysis is a powerful tool for determining genes associated with molecular mechanisms underlying biological processes of interest and for defining the function of a gene in a cell type using bulk-tissue transcriptomic data. GCNs provide insights into gene function in specific cell types by detecting gene clusters (i.e. modules) enriched for cell type markers. By applying the ‘guilt-by-association’ (GBA) heuristic (van Dam et al., 2017; Wolfe et al., 2005) to GCNs, all genes in a cell-type enriched module are then predicted to relate to a single cell type within which, they share the same function.

However, there is ample evidence to suggest that a single gene may have different biological functions in different cellular contexts. For example, the tumor suppressor gene, *TP53*, which encodes tumor protein p53 has different roles depending on its interaction partners and consequently is implicated not only in DNA damage and repair, but also in the initiation of apoptosis and senescence (Gillis and Pavlidis, 2011). Yet current GCN analyses cannot capture such complexity even if it is reflected in transcriptomic data because commonly used forms of GCN analysis assign a single gene to a single module (see Supplementary Methods, Section S2.1., for a detailed discussion on this). This is a significant limitation, especially when considering tissues with high cellular heterogeneity such as human brain tissue, where cellular context is likely to be of key importance to the understanding of gene function. Thus, elucidating the different roles of critical genes, while accounting for different contexts, such as expression in different cell types, could reveal new insights into the comprehension of gene function (Eating Disorders Working Group of the Psychiatric Genomics Consortium *et al.*, 2020; Hekselman and Yeger-Lotem, 2020; Muratore et al., 2017; Reynolds et al., 2019).

While cell-specific transcriptomic analyses may provide a means of addressing that issue, there are significant challenges associated with the construction of co-expression-based approaches in this context. Although single-cell technology is evolving rapidly, the gene expression data generated remains sparse in nature, with low sensitivity on the reads per gene and cell types detected when using single-cell and single-nucleus RNA-sequencing approaches, respectively. Other issues include drop-outs or transcriptional bursting (i.e. the time depending variation of transcription activity) (Buettner et al., 2015; Handley et al., 2015; Haque et al., 2017; Hicks et al., 2018; Kolodziejczyk et al., 2015; Zhu et al., 2018). Furthermore, cell-specific human brain gene expression datasets generated from large numbers of individuals are rare due to the associated costs, and are largely limited to single-nucleus RNA-sequencing (snRNAseq) data as tissue is primarily sampled from post-mortem tissue. These snRNAseq datasets not only tend to be particularly sparse, but also have systematic biases, such as the under-representation of genes expressed within the neuropil (i.e. the synaptically enriched area of the central nervous system). To overcome some of these challenges, alternative techniques have been developed to study specific cell types in bulk tissue, for example by deconvoluting the specific contribution of each cell type to gene expression (Baron et al., 2016; Dong et al., 2020; Newman et al., 2015, 2019; Tsoucas et al., 2019; Wang et al., 2019).

**Fig. 1.**
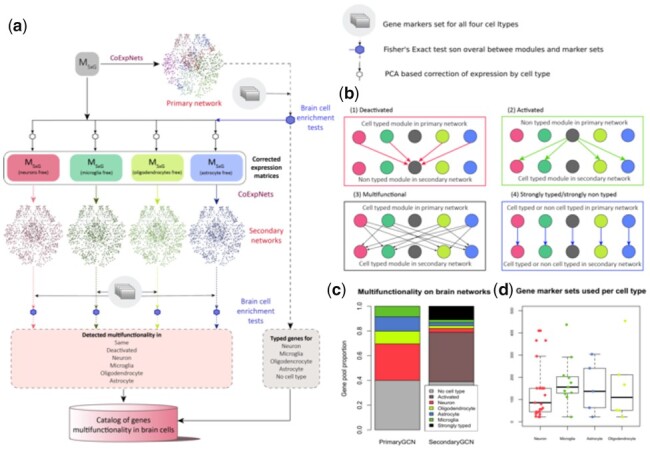
(**a**) GMSCA generates a list of triplets <gene, cell type, function> for the genes included in the initial gene expression profiling matrix, MSxG. First, a WGCNA+k-means co-expression network called the primary network is created. Its modules are then tested (Fisher’s Exact Test, FET) for enrichment of cell type markers, in this case with brain cell type marker sets for neurons (in red), microglia (in green), oligodendrocytes (in light green) and astrocytes (in blue). Those modules with a clear signal (FET *P* < 0.05 on just a single cell type) are selected and their corresponding cell signal removed (see Section 2) from expression to generate a new M’SxG. GMSCA creates a new co-expression network (these are called secondary) for each expression matrix and annotates their modules in the same way. Cell-type enriched modules in both primary and secondary networks generate as many triplets (gene, cell type, function) as genes in the module. (**b**) Any gene found in a cell type enriched module is tagged by GMSCA as ‘typed’. When a gene in a primary co-expression network shifts from a cell-type enriched module to a non-cell-type enriched module in the secondary co-expression network, we say the gene is deactivated (red arrows). When it goes from a non-cell-type enriched module to a cell-type enriched module, we say the gene is activated (green arrows). A gene is multifunctional when it goes from a cell-type enriched module to a module with a different cell type enrichment (black arrows). A gene is strongly typed when it goes from a cell-type enriched module to another module with the same cell-type enrichment. It is strongly non-typed when the primary module and secondary module are both non-cell-type enriched. (**c**) An average primary co-expression network tags 37% of genes as non-typed, another 30% as neuronal, 10% as oligodendrocytic, 13% as astrocytic and 8% as microglial. In a secondary network, 37% of non-typed genes become typed and 42.5% of typed genes become deactivated. Also, as 9.1% of the genes are tagged as pleiotropic in a single network, we can gain up to 36.4% annotations of pleiotropy with all four secondary networks GMSCA creates in this case. (**d**) Number and size of gene markers set used by GMSCA for each cell type, in this article

Thus, the development of tools to maximize the value of the large quantities of deeply sequenced and publicly available human brain bulk-tissue transcriptomic datasets remains important. In this study, we develop a new method to investigate gene multifunctionality in bulk-tissue transcriptomic datasets. Here, we define multifunctionality as the association of a gene to multiple biological functions within a tissue as a result of its different cellular contexts. Particularly, we are primarily interested in the cell type context of genes. And our aim is to uncover different cell types and functions for the same genes and in this way opening new ways to study diseases with a genetic basis. We propose Gene Multifunctionality in Secondary Co-expression network Analysis (GMSCA) to investigate gene multifunctionality on gene expression profiling from bulk tissue (see Fig. 1a and b). GMSCA is applicable when single-cell based data is not available yet but also in settings where there are paired bulk-tissue and single-cell samples. Importantly, GMSCA has been developed not to focus on producing estimates of each cell contribution to gene expression but on predicting gene function in each cell type the gene is expressed in. This process involves two steps. Firstly, GMSCA constructs a primary gene co-expression network (PGCN) from a gene expression matrix to produce a primary set of triplets <gene, cell type, function> from the annotated PGCN (see Supplementary Section S2.2. of Supplementary Methods). All genes found in those triplets are said to be typed. Secondly, GMSCA creates secondary gene co-expression networks (SGCN) for each of the target cell types. For each cell type and modules enriched for that cell type within the PGCNs, GMSCA removes the contribution of that cell type from the expression matrix. GMSCA now constructs, with the newly created gene expression matrix (which includes all the original gene pool), a SGCN and extracts new triplets <gene, cell type, function> (see Fig. 1c and Supplementary Methods). When a gene was not identified within a triplet in the PGCN, but now appears in any triplet from the SGCNs, we say the gene is activated. If, on the contrary, it appeared within a triplet generated from the PGCN but not within any of the SGCN triplets, it is termed deactivated (see Fig. 1b for details).

GMSCA increases the number of triplets obtained by 46.73% on average compared to conventional GCN, so notably increasing its utility. Therefore, secondary co-expression networks can augment existing tissue deconvolution approaches as they generate additional functional annotation of different cell types. These new functional annotations include multiple annotations for single genes (i.e. they shape gene’s multifunctionality), creating new applications for this well-established analysis.

## 2 Materials and methods

We created three network families in the form of R packages as resources: CoExp10UKBEC, CoExpROSMAP and CoExpGTEx. Networks were created with WGCNA and refined with k-means with CoExpNets R package (Bot**í**a et al., 2017).

CoExp10UKBEC comprises GCNs created from confirmed non-pathological human brain samples of 10 different brain areas. Samples were profiled for gene expression with Affymetrix Human Exon v2. Microarrays. Identical gene pool was used to construct co-expression networks [details of GCN construction here (Forabosco et al., 2013)].

ROSMAP is a transcriptomics human frontal cortex resource with RNA-seq based gene expression profiling for 640 samples, corrected for batch effect using ComBat. Age, sex, RIN and PMI (Post-Mortem Interval) were regressed out of individual gene expression. GCNs were constructed from residuals. The 640 samples were arranged into four different groups to create 4 GCNs. One with all samples and three depending on neuritic plaques deposition post-mortem diagnostic (a group for values 1, a second group for values 2 and 3 and a third group for values 4) with 200, 158 and 221 respectively.

We developed 13 co-expression networks for the 13 available brain areas in GTEx RNA-seq samples. We corrected samples for batch effect with ComBat. Then, we generated surrogated variables to model unknown effects in data and regressed those, age, sex, PMI and RIN from the overall gene expression. GCNs were constructed from residuals. See the Supplementary for the effect of sample size in GCN construction.

The procedure for creating both PGCNs and SGNCs is identical. The smooth parameter that satisfies scale free topology is found. An adjacency matrix is calculated and the subsequent topology overlap matrix (TOM) is obtained. The clustering is performed using 1-TOM as distance. Gene clusters are obtained by the cuTree algorithm with 100 as the minimum number of genes. A refinement step based on the k-means algorithm with 50 iteration steps is applied afterwards. Genes are ranked within the modules through the Pearson correlation of their expression and the 1st principal component of gene expression of the whole module, the module membership, MM. Gene clusters are annotated with function using gProfiler [see details here (Bot**í**a et al., 2017)]. All networks are annotated for cell type as follows.

### 2.1 Module annotation for function and cell type

To annotate network modules for specific brain cell types, we tested modules for brain specific gene markers for the four main brain cell types **(**neuron, microglia, astrocytes and oligodendrocytes) (see Fig. 1d) using manually curated cell marker datasets found at the CoExpNets package. We redesigned the original Fisher’s Exact test employed in CoExpNets to assess the significance of the overlap between genes at modules and genes at marker sets to account for module size as there is significant association between network module size and -log10() transformation of *P*-values from the tests in all PGCNs, mean R^2^ 0.28, min 0.17, max 0.45 (Supplementary Fig. S4a and b). After applying Bonferroni correction, to account for multiple testing, we regressed out gene set size effect from *P*-values significance (Supplementary Fig. S4c and d, Supplementary Methods S2.3.2.) reducing the number of positive tests from an average of 22 per network to just a mean of 11.03. Note that GMSCA is not restricted to either to the cell types considered here, nor the gene marker sets from CoExpNets. It can be tailored to any tissue and its corresponding cell types of interest provided that there are suitable cell-type-specific markers.

### 2.2 Human phenotype ontology enrichment analysis

Human Phenotype Ontology (HPO) terms associated with each human gene were downloaded from HPO (Köhler et al., 2019). To determine the enriched Human Phenotype Ontology (HPO) terms we have selected the relevant overrepresented phenotypes in each module, these are phenotypes that are in at least 2% of the genes from the module and Fold Change (FC) is a measure of how many times that phenotype is more likely to be found in the module than by chance (HPO ontology*)*.

### 2.3 Secondary co-expression networks generation

After generating the PGCN of a gene expression matrix, GMSCA detects modules enriched for the cell types of interest. For each gene in a module enriched for cell type ct, GMSCA generates a prediction triplet <gene, ct, function> where *function* refers to the Gene Ontology based annotation for all genes in the module. Then it builds SGCNs by applying a transformation to the gene expression of genes at modules enriched for the cell types of interest. GMSCA’s model of gene expression for any gene g as follow*s*
 

$$e=\sum_{c\in CT\;}^{\;}\alpha_{c}x_{c}+\;\beta \;x_{0},$$

where *e* is the gene expression for a specific gene, CT is the set of all cell types considered of interest for the tissue, *x*_c_ is the particular contribution per sample of cell type c to the gene, and *x*_o_ represents other unknown factors contributing to the expression. GMSCA assumes this additive model to decompose the gene expression matrix of each cell type enriched module as follows. Let us suppose that module m is enriched for a particular cell type. GMSCA removes the contribution of that cell type to gene expression from module m by starting with the *M*_S,__m_ matrix (i.e. gene expression of genes in module m for all samples *S*), it then obtains the set of PCAs to explain 90% of the variance, and compounds a new matrix with them, *T*_m,__m,_ the transformed gene expression matrix. It then removes the first PCA from that matrix to create T’_m-1, m-1_. Note that the 1st PCA is assumed to be the cell type contribution to gene expression that we remove. T’_m-1, m-1_ is finally used to reconstruct the original expression, using all PCA axes but the 1st. The current matrix, M’_S, m_, is now free of the detected cell type contribution. This matrix is then used by GMSCA to create a new GCN that we call secondary, i.e. SGCN. GMSCA constructs, in this way, one SGCN for each cell type. Note that it is extremely rare to find gene modules enriched for more than one cell type. And in those cases, the enrichment of one of the cell types is almost marginal, while the other is highly significant. GMSCA drops out the marginal signal.

### 2.4 Cell-type-specific gene expression estimates for Barres

We downloaded Supplementary Table S4 from Zhang et al. (2016) which contains expression levels for neurons, mature astrocytes, microglia and oligodendrocytes. Within each cell type, we averaged all available values for each gene. We scale and log transform that matrix. Then we generate a boolean matrix of specific gene expression in cell types, M_g x ct_ with genes in rows and cell types in columns such that M[i, j] is set to TRUE when gene i has greater expression than the mean expression from the whole expression matrix.

To assess the multifunctionality prediction overlap between Barres data and GMSCA, we looked at GMSCA predictions from PGCNs and SGCNs, and assessed the overlap in the corresponding cell type at the Barres’ data. We report a Fisher’s exact test on this overlap. In each comparison, we remove known gene markers to avoid optimistic and unfair overlap assessments.

### 2.5 Cell-type-specific gene expression estimates for sc-ROSMAP

We downloaded the sc-ROSMAP filtered count matrix from the Synapse Web site. In sc-ROSMAP, we can find 48 samples of human frontal cortex tissue arranged into 24 Alzheimer’s disease cases and 24 controls. We used control, dropped all zero count cell samples and generated a Boolean matrix of gene expression in neurons, microglia, mature astrocytes and oligodendrocytes, M_g x ct_ with genes in rows and cell types in columns such that M[i, j] is TRUE when gene i has a fold change of 3 of expression in cell type j, with respect to the overall mean expression within that gene and cell type.

We assess the significance (Fisher’s exact test) of the overlap between all four ROSMAP network predictions and single cell observations. We removed known gene markers to avoid optimistic and unfair overlap assessments and aggregated excitatory and inhibitory neurons into a single type.

### 2.6 Using EWCE to replicate cell-specific expression enrichment from GSMCA predictions

EWCE was used to assess whether multifunctional predictions from GMSCA show any enrichment in expression in equivalent cell types from mouse. We tested each predicted cell type gene set from GMSCA for enrichment in the equivalent cell type at EWCE with tye bootstrap.enrichment.test() from the EWCE package, we converted human genes in our networks into MGI symbols using the mouse_to_human_homologs data table from EWCE. Gene markers were removed from the analysis. We used all MGI genes at the mouse_to_human_homologs table as the background gene set and 10 000 permutations for each test. Results with *P* < 0.05 were reported as significant.

## 3 Results

To investigate the utility of GMSCA for the prediction of gene multifunctionality in brain, we used 27 co-expression networks from three brain-derived gene expression datasets (see Supplementary Table S1), including 13 bulk RNA-seq networks from GTEx’s (The GTEx Consortium *et al.*, 2015) brain regions and 4 bulk RNA-seq networks from the ROSMAP (Bennett et al., 2012a, b) project, all variations of frontal cortex samples. Further, we reused 10 bulk AffyExon Array networks derived from UKBEC’s 10 brain regions (Ramasamy *et al.*, 2014). We refer to them as the GTEx, ROSMAP and UKBEC network families respectively.

The average gene pool for the three network families is 18 682 and the average number of modules (clusters) is 30. As GMSCA uncovers multifunctionality, it focuses on modules enriched for markers of the cell types of interest. The average cell type enriched module size is 622 genes. Note that a percentage of those genes are cell type markers themselves; on average 158 genes, i.e. 25% of the whole module. Therefore, 75% of the genes in those modules generate new <gene, cell type, function> triplets in that cell type. Overall, the average PGCN generates more than 4500 of those prediction triplets. We focus on the major cell types in brain: neurons, microglia, astrocytes and oligodendrocytes (see Fig. 1d). We found that 44% of the PGCN modules enriched for markers of the major cell types were tagged as neurons, 21% as microglia, 18% as astrocytes and 17% as oligodendrocytes. Thanks to this approach, we gained the capability of studying gene multifunctionality by discovering new gene multifunctionality in specific cells with an additional 46.73% of the overall gene pool. 37% of the gene pool refers to newly activated genes and 9.73% are additional cell types for genes that become multifunctional (see Fig. 1c).

### 3.1 GCN modules enriched for cell type markers are reliable

**Fig. 2.**
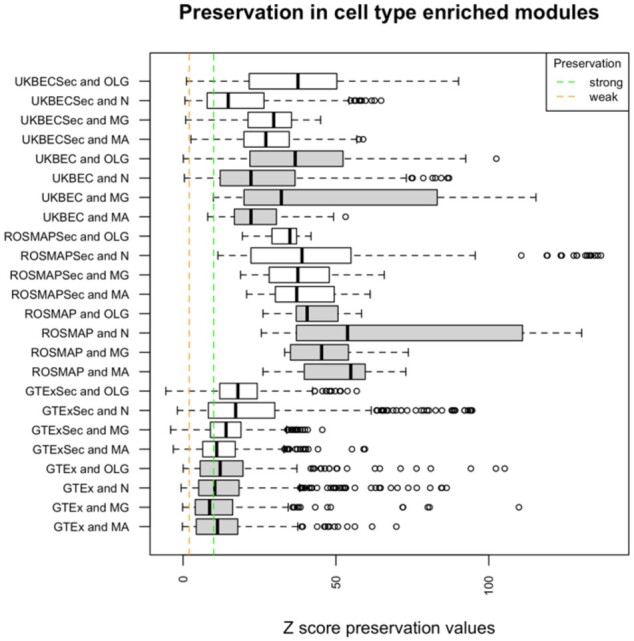
distribution of preservation values for all cell-type enriched modules as detected by GMSCA, for primary (in grey) and secondary (in white) networks. Each box plot corresponds to all modules within a family found to be enriched for the indicated cell type (MA for mature astrocytes, MG for microglia, N for neuron and OLG for oligodendrocytes). Families ending with ‘Sec’ refer to secondary network modules. The vertical green dashed line marks the strong preservation limit (modules are easily replicable in other brain tissues and therefore highly reliable). The vertical orange dashed line marks the weak preservation limit (modules show signs or preservation and therefore some evidence of reliability). All preservation tests are performed within each network family. 98.1% of the preservervation tests sustain network reliability in UKBEC and 95.5% in GTEx. All tests yield strong preservation within the ROSMAP family

Predictions made by GMSCA come from cell marker enriched modules. Then we assessed whether those modules were replicable (i.e. preserved), and therefore reliable, across brain areas. If a gene module is preserved in a similar tissue, then the module is credible and replicable. If the module is not preserved, it might be due to two reasons. Either the module is specific for that tissue, and hence reliable, or the module is not reliable, i.e. most genes are found there just by chance. We used WGNCA’s preservation analysis based on the estimation of a *Z* statistic called *Z* summary (see Langfelder and Horvath, 2008 for a detailed explanation). Values of Z over 10 for a module suggest strong module preservation. Values over 2 suggest some preservation. Modules with Z under 2 are not preserved at the tissue tested (see Fig. 2).

We first tested GTEx PGCNs’s modules enriched for cell type-specific markers for preservation across the remaining GTEx PGCNs. This resulted in 560 preservation tests. 8.6% of those tests were negative (i.e. Z < 2) suggesting that those modules were not preserved in the remaining brain PGCNs, while 40.4% of the tests showed signs of preservation (Z ≥ 2) and 50.8% showed strong preservation (Z ≥ 10). We performed 630 tests at UKBEC networks and 1.9% were negative. 13.3% pointed to weak preservation and the remaining 84.7% indicated strong preservation. All ROSMAP target modules were very well preserved as we may expect: one of the PGCNs includes all ROSMAP samples, and the other three include subsets of the samples used within that PGCN.

Next, we assessed the 52 GTEx SGCNs (each PGCN generates four new GCNs) for preservation. This resulted in 5157 tests being performed of which 4.4% were negative (a cell type enriched module was not preserved in a GTEx tissue), 25.9% implied weak preservation and 69.6% yielded strong preservation. We performed the same analysis for the 40 UKBEC SGCNs. In this case, 2310 preservation tests were performed and 1.12% of the tests were negative, whereas 17.7% of the tests showed signs of preservation and 81.12% showed strong preservation. All preservation tests in ROSMAP SGCNs showed strong preservation.

Part of the difference in non-preserved modules between GTEx and UKBEC can be explained due to PGCNs module size. The Z preservation value correlates positively with module size (R^2 0.38, Supplementary Fig. S1) (Langfelder et al., 2011). And maximum module size in GTEx PGCNs is 1663, and 2953 in UKBEC. Additionally, we wanted to further investigate whether the non-preserved GTEx modules could have had some biological meaning. We performed a functional enrichment analysis on the genes at those modules, based on the Gene Ontology, REACTOME and KEGG pathway databases using gProfileR R package (Reimand et al., 2007). On average, we obtained 164 annotation terms per non-preserved module, suggesting these modules were biologically relevant. Their lack of preservation may indicate that these modules were specific to their tissue.

### 3.2 GMSCA multifunctional predictions replicate well in cell-type-specific datasets

To assess whether GMSCA multifunctionality predictions replicate in cell-specific brain datasets we considered two cell-specific gene expression datasets in human brain cortex, sc-ROSMAP and Barres Lab data, which are different in nature and a mouse brain expression dataset (Zeisel et al., 2018) through the EWCE (Skene and Grant, 2016) tool.

One of the human datasets is sc-ROSMAP (Mathys et al., 2019), from single cell transcriptomics in a fraction of bulk-RNAseq ROSMAP paired brain cortex samples (24 AD cases and 24 controls). The other is based on immunopanning of temporal lobe cortex samples (Mathys *et al.*, 2019; Zhang *et al.*, 2016) from Barres Lab. This involved purification of specific cell types using cell surface markers followed by gene expression profiling on these purified cell types. As we already noted, sc-ROSMAP and Barres’ datasets are of different nature but both were reduced to a list of cell-type-specific genes for each of the cell types we are using in this article (see Section 2). These lists are used then to compare with the cell multifunctionality predictions generated by GMSCA. Cell markers within GMSCA are removed from the analysis to avoid optimistically biased results.

Multifunctionality predictions from all four ROSMAP PGCNs yielded significant overlap with sc-ROSMAP data (see Supplementary File S2). For example, in the NotAD network the overlaps are as follows: Fisher’s Exact Test (FET): *P* < 4.27E-36 for neurons, *P* < 3.27E-46 for oligodendrocytes, 0.017 for microglia and 2.94E-42 for astrocytes. SGCN predictions generated overlaps with the following significance: FET *P* < 2.02E-36 for neurons, *P* < 2.4E-7 for oligodendrocytes, *P* < 2.3E-3 for Microglia and *P* < 1.28E-3 for astrocytes.

Barres Lab based predictions were assessed for overlap with our cortex networks (see Supplementary File S2). Focusing, for example, on the same tissue used within the Barres paper, temporal lobe (the 10UKBEC TCTX GCN), GMSCA generated highly significant overlaps in primary predictions with *P* < 4.51E-67 for neurons, *P* < 1.94E-31 for oligodendrocytes, *P* < 7.2E-13 for microglia and *P* < 1.01E-84 for astrocytes. Secondary predictions yielded significant overlaps for neurons (*P* < 1.19E-73), oligodendrocytes (*P* < 4.09E-42) and astrocytes (*P* 2.02E-12), but not for microglia (*P* 0.91).

GMSCA predictions were assessed for replication on a different-species and more comprehensive single-cell brain transcriptomic dataset, through EWCE (Expression Weighted Cell Enrichment). This tool enables easy integration with mouse brain transcriptomics from 19 brain regions (Zeisel et al., 2015) and can be used to test whether a given gene list provided by the user presents higher expression levels in each cell type from the reference dataset than expected by chance. We tested all cell type predictions from PGCNs and SGCNs to assess whether EWCE recapitulated similar enrichments. This analysis showed that 100% of the GMSCA gene sets predicted to be functional in a given cell type were also found to be significantly enriched in expression by EWCE in the corresponding cell type (see Section 2, Supplementary File S5*)*.

### 3.3 GMSCA multifunctional predictions show concordance across the three network families in cortex-like tissues

As we used three brain network families, we could compare same-tissue networks between families to assess the agreement of predictions across their networks. Cortex is the only tissue present in all three families but also putamen and substantia nigra in GTEx and UKBEC (remind that ROSMAP is only frontal cortex). We assessed the significance of each pairwise overlap between (gene, cell-type) predictions from both primary and secondary networks, for each cell type and pair of frontal cortex tissues. All Fisher’s exact tests performed on the pairwise overlaps were highly significant, being the higher *P*-value of 1.3E-318 (see Supplementary File S4 and Supplementary Fig. S2).

In regard to the putamen brain area, 35% of the neuron predictions made from the UKBEC putamen networks (primary and secondary), are also found as neuron in the GTEx putamen networks. Note that the putamen UKBEC PGCN generates no microglia related predictions (it generates 2686 predictions in the SGCN though). The agreements for oligodendrocytes and astrocytes are 22.01% and 49.76%, respectively. For the substantia nigra brain area, the numbers for neuron, microglia, oligodendrocytes and astrocytes are 28.62%, 35.98%, 56.26% and 42.91% respectively (see Supplementary File S4 and Supplementary Fig. S3).

### 3.4 GMSCA links the SNCA gene to oligodendrocytes

The *SNCA* gene encodes the alpha-synuclein protein, located at the long arm of chromosome 4 at position 4q22.1. Mutations in this gene cause Parkinson disease (PD) (Appel-Cresswell et al., 2013; Krüger et al., 1998; Lesage *et al.*, 2013; Polymeropoulos, 1997; Zarranz et al., 2004). *SNCA* is also linked to other neuro-degenerative diseases, mainly Alzheimer’s disease (Hashimoto and Masliah, 1999). The alpha-synuclein protein is involved in the regulation of neurotransmitter release, synaptic function and plasticity of dopaminergic neurons (Cheng et al., 2011). *SNCA* is also are associated with neuronal dysfunction. Therefore, it has been traditionally seen as highly relevant to neurons. Thanks to applying GMSCA to our networks, the generated multifunctionality predictions included an association of *SNCA* to oligodendrocytes in 4 SGCNs, see below (Supplementary File S1).

**Fig. 3.**
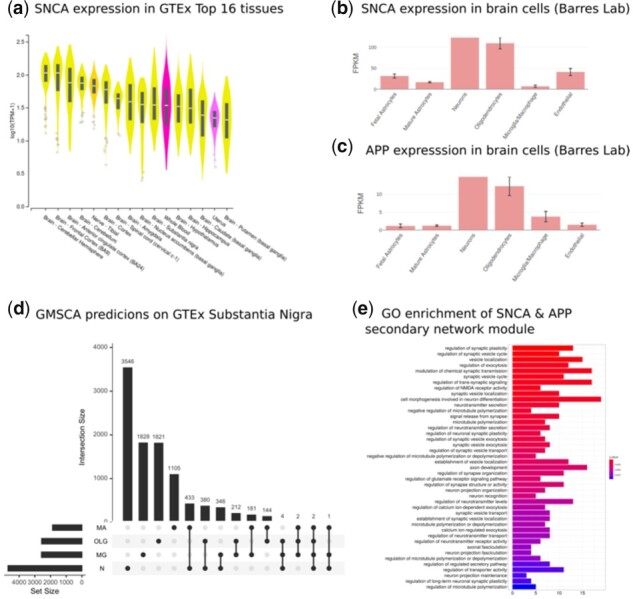
(**a**) SNCA is predominantly expressed in brain, as the violin plots show. All 13 brain tissues are within the top 16 GTEx tissues expressing SNCA. (**b**) Immunopanning data on brain cell expression from Barres Lab shows SNCA predominantly expresses in neurons and oligodendrocytes. GMSCA tags SNCA as neuronal and oligodendrocytic in the substantia nigra, putamen and hippocampus. (**c**) APP, genetically linked to Alzheimer, is another interesting gene found in the same module as SNCA. Barres Lab’s data confirms it is predominantly expressed in neurons and oligodendrocytes. GMSCA tags it as neuronal and oligodendrocytic. (**d**) UpSet plot (UpSet R package) on the genes from the predictions by GMSCA on the GTEx substantia nigra samples. SNCA and APP are found at the intersection between N (neurons) and OLG (oligodendrocytes) with 378 genes more. (e) gProfileR enrichment annotation for the secondary network module midnightblue in which we find SNCA and APP appearing together

*SNCA* is predominantly expressed in brain tissue (Fig. 3a), according to GTEx control samples bulk expression data V8 (The GTEx Consortium *et al.*, 2015). Moreover, *SNCA* is mainly expressed in neurons and oligodendrocytes (Zhang *et al.*, 2016) in the human cortex (Fig. 3b and c), as the Barres Lab data shows. In fact, *SNCA* was found in neuron-enriched modules in 10 out of the 13 GTEx PGCNs, all 4 ROSMAP networks and in 8 out of the 10 UKBEC networks. The only alternative cell type linked to *SNCA* is oligodendrocytes through the GTEx SGCNs of substantia nigra, putamen and hippocampus and the UKBEC temporal cortex.

The substantia nigra is a central tissue in PD. Therefore we focused the subsequent analysis on this tissue (Fig. 3d). *SNCA* is found in the orangered2 module of the PGCN (module membership, MM = 0.84, see Section 2). That module is enriched for dopaminergic neuron markers (*P* < 4.59E-25) and GO terms like catecholamine and dopamine biosynthetic processes (GO:0042423 *P* < 1.1E-5; GO:0042416 *P* < 9.9E-5), locomotory behavior (GO:0007626 *P* < 4.1E-4), neurotransmitter transport (GO:0006836 *P* < 1.9E-5), axonogenesis (GO:0007409 *P* < 6.4E-4), dopaminergic neuron differentiation (GO:0071542 *P* < 8.0E-3) and ferric ion transport (GO:0015682 *P* < 4.6E-4). Dopaminergic neuronal loss is the hallmark of PD. Moreover, the aforementioned biological processes are highly relevant to PD (Aggarwal *et al.*, 2019; Nutt et al., 2004; Sawada et al., 2013). Interestingly, the darkorange2 module includes six more genes linked to PD (Supplementary Table S1). All this evidence suggests this network module is linked to PD.

When we applied GMSCA to create the corresponding SGCN, *SNCA* is found in the substantia nigra midnightblue module (MM = 0.53), which is enriched for markers of oligodendrocytes (*P* < 1.48E-4). This finding is replicated using the putamen and hippocampus co-expression networks, belonging to the same network family, GTEx. Moreover, themidnightblue module shared 27 genes with the *SNCA* SGCN module in putamen (Fisher’s exact test on the overlap significant, *P* < 5.3E-11) and 45 with the *SNCA* SGCN module at the hippocampus, *P* < 2.2E-16, suggesting function similarity of these modules across the three tissues linking *SNCA* to oligodendrocytes. The midnightblue module (Fig. 3e) was enriched for the following GO terms: synaptic plasticity (GO:0048167 *P* < 1.3E-4), neuronal plasticity (GO:0048168 *P* < 1.9E-3), regulation of synapse organization (GO:0050807 *P* < 3.5E-3), axonal fasciculation (GO:0007413 *P* < 7.2E-3), neuron projection fasciculation (GO:0106030 *P* < 7.2E-3), axon development (GO:0061564 *P* < 3.5E-3), neuron recognition (GO:0008038 *P* < 4.1E-3), regulation of neurotransmitter levels (GO:0001505 *P* < 5.5E-3), microtubule polymerization (GO:0046785 *P* < 1.4E-3), regulation of glutamate receptor signalling pathway (GO:1900449 *P* < 3.6E-3) and synaptic vesicle exocytosis (GO:0016079 *P* < 2.1E-3). Note that BP terms like ‘regulation of neurotransmitter levels’ and ‘synaptic vesicle exocytosis’ are processes found in the orangered2 module of the substantia nigra PGCN. Terms specific to the SGCN module, and also linked to oligodendrocytic activity, were: regulation of synapse organization (Eroglu and Barres, 2010), regulation of glutamate receptor signaling pathway (Gautier et al., 2015), axon development, axonal fasciculation, neuron projection fasciculation, neuron recognition or microtubule polymerization [50]. Interestingly, the midnightblue module included *APP* (MM = 0.84). Mutations in *APP* increase the risk of Alzheimer’s disease (Köhler *et al.*, 2019). *APP*’s expression pattern in specific brain cells is similar to *SNCA*’s pattern, mainly expressed in neurons and oligodendrocytes (see Fig. 3c). The *APP* gene has been reported to have a role in regulating axonal myelination in oligodendrocytes (Truong et al., 2019).

A gene based analysis of the phenotypes linked to the midnightblue module (see Section 2) uncovered HPO terms like Dementia (HP:0000726 fold change 2.55), Myoclonus (HP:0001336 FC 2.13), Variable Expressivity (HP:0003828 FC 1.85), Anxiety (HP:0000739 FC 1.65), Intellectual severe disability (HP:0010864 FC 1.56), Depressivity (HP:0000716 FC 1.24), Tremor (HP:0001337 FC 1.22), Ataxia (HP:0001251 FC 1.2) and Dystonia (HP:0001332 FC: 1.16).

## 4 Discussion

We propose GMSCA (Gene Multifunctionality Co-expression Analysis), as a method and software tool to uncover additional co-expression profiles for genes, apart from those we obtain with conventional co-expression analyses. GMSCA significantly enhances the power of GCN analyses, delivering the capability to study gene multifunctionality in specific cells, as models of gene co-expression in bulk tissue. GMSCA needs the same inputs as conventional co-expression analysis on bulk tissue such as gene expression profiles, gene marker sets for the cell type under study and gene sets enrichment analysis tools as gProfileR for pathway or GO based module annotation. This article’s experiments are based on brain samples. Therefore, the SGCN obtained were focused on neurons, microglia, astrocytes and oligodendrocytes cell types. Interestingly, this approach increased the number of predictions of gene multifunctionality in specific cells with an additional 46.73% of the overall gene pool.

To demonstrate the potential of this method, we firstly assessed the reliability of GMSCA prediction triplets by means of its application to three network families for three different transcriptomics-based projects, UKBEC, GTEx and ROSMAP using WGCNA’s preservation analysis. We demonstrated that the cell-type enriched modules that GMSCA used to generate multifunctionality predictions were stable, including those in SGCNs. Part of the difference we obtained in non-preserved modules between GTEx and UKBEC could be explained by PGCNs module size.

Secondly, we showed a high level of replication of predictions in cell-type-specific external datasets. As we already noted, sc-ROSMAP and Barres’ datasets are of different nature but both were reduced to a list of cell-type-specific genes for each of the cell types. Multifunctionality predictions from all four ROSMAP PGCNs yielded significant overlaps with the single nucleus ROSMAP dataset. We assessed prediction replication in Barres Lab data by looking at how our cortex networks’ predictions replicated in Barres cell specific gene expression profiles. All cell types but microglia showed highly significant levels of agreement. Moreover, thanks to the EWCE tool we were capable of assessing GMSCA predictions on mouse single-cell transcriptomics. 100% of both PGCN and SGCN gene sets yielded significant expression enrichment in equivalent brain cells.

Then, we tested the level of agreement in GMSCA predictions on the same tissues across the three networks families (frontal cortex tissue across the three families and substantia nigra and putamen tissues across GTEx and UKBEC) and demonstrated that agreement was high. We compared same-tissue networks between families and assessed the agreement level of predictions for cell marker enriched modules across their networks, in PGCNs and SGCNs, for preservation across brain areas.

We used this SGCN to get details about the biological function of *SNCA* within specific cell types, such as how relevant *SNCA* was in the corresponding module and what genes *SNCA* co-expressed with, e.g. APP linked to dementia and Alzheimer. In this study, *SNCA* was found in oligodendrocytes through the GTEx SGCNs of substantia nigra, putamen and hippocampus and through the UKBEC SGCNs of temporal cortex. The relationship of *SNCA* and oligodendrocytes is supported by previous studies that have observed alpha-synuclein-containing inclusions (‘coiled bodies’) in oligodendrocytes in parkinsonian brains (Wakabayashi et al., 2000). Recently, the link between oligodendrocytes and Parkinson’s disease was reinforced by integrating GWAS results with single-cell transcriptomic data (Eating Disorders Working Group of the Psychiatric Genomics Consortium *et al.*, 2020) through testing the genes tagged by the GWAS outcome into the EWCE tool. Thus, GMSCA has the potential to shed light on gene function within specific cell types and molecular processes, particularly in a disease context. To conclude, we would like to emphasize that the utility of GMCSA is not limited to brain tissue, but this method could be easily tailored to address questions around gene multifunctionality in any other tissue assuming the availability of expression profiling data and high quality, relevant cell-specific markers.

## Supplementary Material

btab175_Supplementary_Data

## References

[btab175-B1] Aggarwal A. et al (2019) A locomotor assay reveals deficits in heterozygous Parkinson’s disease model and proprioceptive mutants in adult *Drosophila*. Proc. Natl. Acad. Sci. USA, 116, 24830–24839. 31748267 10.1073/pnas.1807456116 PMC6900508

[btab175-B2] Appel-Cresswell S. et al (2013) Alpha-synuclein p.H50Q, a novel pathogenic mutation for Parkinson’s disease: α- Synuclein p.H50q, A Novel Mutation For Pd. Mov. Disord., 28, 811–813. 23457019 10.1002/mds.25421

[btab175-B3] Baron M. et al (2016) A single-cell transcriptomic map of the human and mouse pancreas reveals inter- and intra-cell population structure. Cell Syst., 3, 346–360.e4. 27667365 10.1016/j.cels.2016.08.011 PMC5228327

[btab175-B4] Bennett A. et al (2012a) Overview and findings from the religious orders study. Curr. Alzheimer Res., 9, 628–645. 22471860 10.2174/156720512801322573 PMC3409291

[btab175-B5] Bennett A. et al (2012b) Overview and findings from the rush memory and aging project. Curr. Alzheimer Res., 9, 646–663. 22471867 10.2174/156720512801322663 PMC3439198

[btab175-B6] Botía J.A. et al (2017) An additional k-means clustering step improves the biological features of WGCNA gene co-expression networks. BMC Syst. Biol., 11, 47. 28403906 10.1186/s12918-017-0420-6 PMC5389000

[btab175-B7] Bryois J. et al; 23andMe Research Team. (2020) Genetic identification of cell types underlying brain complex traits yields insights into the etiology of Parkinson’s disease. Nat. Genet., 52, 482–493. 32341526 10.1038/s41588-020-0610-9 PMC7930801

[btab175-B8] Buettner F. et al (2015) Computational analysis of cell-to-cell heterogeneity in single-cell RNA-sequencing data reveals hidden subpopulations of cells. Nat. Biotechnol., 33, 155–160. 25599176 10.1038/nbt.3102

[btab175-B9] Cheng F. et al (2011) The role of alpha-synuclein in neurotransmission and synaptic plasticity. J. Chem. Neuroanat., 42, 242–248. 21167933 10.1016/j.jchemneu.2010.12.001

[btab175-B10] Dong M. et al (2020) SCDC: bulk gene expression deconvolution by multiple single-cell RNA sequencing references. Brief. Bioinform., 146, 2563–2575. 10.1093/bib/bbz166 PMC7820884 31925417

[btab175-B11] Eroglu C. , Barres B.A. (2010) Regulation of synaptic connectivity by glia. Nature, 468, 223–231. 21068831 10.1038/nature09612 PMC4431554

[btab175-B12] Forabosco P. et al (2013) Insights into TREM2 biology by network analysis of human brain gene expression data. Neurobiol. Aging, 34, 2699–2714. 23855984 10.1016/j.neurobiolaging.2013.05.001 PMC3988951

[btab175-B13] Gautier H.O.B. et al (2015) Neuronal activity regulates remyelination via glutamate signalling to oligodendrocyte progenitors. Nat. Commun, 6, 8518. 26439639 10.1038/ncomms9518 PMC4600759

[btab175-B14] Gillis J. , Pavlidis P. (2011) The impact of multifunctional genes on “Guilt by Association” analysis. PLoS ONE, 6, e17258. 21364756 10.1371/journal.pone.0017258 PMC3041792

[btab175-B15] Handley A. et al (2015) Designing cell-type-specific genome-wide experiments. Mol. Cell, 58, 621–631. 26000847 10.1016/j.molcel.2015.04.024

[btab175-B16] Haque A. et al (2017) A practical guide to single-cell RNA-sequencing for biomedical research and clinical applications. Genome Med., 9, 75. 28821273 10.1186/s13073-017-0467-4 PMC5561556

[btab175-B17] Hashimoto M. , Masliah E. (1999) Alpha-synuclein in Lewy Body Disease and Alzheimer’s Disease. Brain Pathol., 9, 707–720. 10517509 10.1111/j.1750-3639.1999.tb00552.x PMC8098211

[btab175-B18] Hekselman I. , Yeger-Lotem E. (2020) Mechanisms of tissue and cell-type specificity in heritable traits and diseases. Nat. Rev. Genet., 21, 137–150. 31913361 10.1038/s41576-019-0200-9

[btab175-B19] Hicks S.C. et al (2018) Missing data and technical variability in single-cell RNA-sequencing experiments. Biostatistics, 19, 562–578. 29121214 10.1093/biostatistics/kxx053 PMC6215955

[btab175-B20] Köhler S. et al (2019) Expansion of the Human Phenotype Ontology (HPO) knowledge base and resources. Nucleic Acids Res., 47, D1018–D1027. 30476213 10.1093/nar/gky1105 PMC6324074

[btab175-B21] Kolodziejczyk A.A. et al (2015) The technology and biology of single-cell RNA sequencing. Mol. Cell, 58, 610–620. 26000846 10.1016/j.molcel.2015.04.005

[btab175-B22] Krüger R. et al (1998) AlaSOPro mutation in the gene encoding α-synuclein in Parkinson’s disease. Nat. Genet., 18, 106–108. 9462735 10.1038/ng0298-106

[btab175-B23] Langfelder P. et al (2011) Is My Network Module Preserved and Reproducible? PLoS Comput. Biol., 7, e1001057. 21283776 10.1371/journal.pcbi.1001057 PMC3024255

[btab175-B24] Langfelder P. , Horvath S. (2008) WGCNA: an R package for weighted correlation network analysis. BMC Bioinformatics, 9, 559. 19114008 10.1186/1471-2105-9-559 PMC2631488

[btab175-B25] Lesage S. et al; French Parkinson's Disease Genetics Study Group. (2013) G51D α-synuclein mutation causes a novel Parkinsonian-pyramidal syndrome: SNCA G51D in Parkinsonism. Ann. Neurol., 73, 459–471. 23526723 10.1002/ana.23894

[btab175-B26] Mathys H. et al (2019) Single-cell transcriptomic analysis of Alzheimer’s disease. Nature, 570, 332–337. 31042697 10.1038/s41586-019-1195-2 PMC6865822

[btab175-B27] Miller J.A. et al (2010) Divergence of human and mouse brain transcriptome highlights Alzheimer disease pathways. Proc. Natl. Acad. Sci. USA, 107, 12698–12703. 20616000 10.1073/pnas.0914257107 PMC2906579

[btab175-B28] Muratore C.R. et al (2017) Cell-type dependent Alzheimer’s disease phenotypes: probing the biology of selective neuronal vulnerability. Stem Cell Rep., 9, 1868–1884. 10.1016/j.stemcr.2017.10.015 PMC5785690 29153990

[btab175-B29] Newman A.M. et al (2015) Robust enumeration of cell subsets from tissue expression profiles. Nat. Methods, 12, 453–457. 25822800 10.1038/nmeth.3337 PMC4739640

[btab175-B30] Newman A.M. et al (2019) Determining cell type abundance and expression from bulk tissues with digital cytometry. Nat. Biotechnol., 37, 773–782. 31061481 10.1038/s41587-019-0114-2 PMC6610714

[btab175-B31] Nutt J.G. et al (2004) The dopamine transporter: importance in Parkinson’s disease. Ann. Neurol., 55, 766–773. 15174010 10.1002/ana.20089

[btab175-B32] Oldham M.C. et al (2008) Functional organization of the transcriptome in human brain. Nat. Neurosci., 11, 1271–1282. 18849986 10.1038/nn.2207 PMC2756411

[btab175-B33] Polymeropoulos M.H. et al (1997) Mutation in the -Synuclein gene identified in families with Parkinson’s Disease. Science, 276, 2045–2047. 9197268 10.1126/science.276.5321.2045

[btab175-B34] Ramasamy A. et al; North American Brain Expression Consortium. (2014) Genetic variability in the regulation of gene expression in ten regions of the human brain. Nat. Neurosci., 17, 1418–1428. 25174004 10.1038/nn.3801 PMC4208299

[btab175-B35] Reimand J. et al (2007) g: profiler—a web-based toolset for functional profiling of gene lists from large-scale experiments. Nucleic Acids Res., 35, W193–W200. 17478515 10.1093/nar/gkm226 PMC1933153

[btab175-B36] Reynolds R.H. et al; International Parkinson’s Disease Genomics Consortium (IPDGC), System Genomics of Parkinson’s Disease (SGPD). (2019) Moving beyond neurons: the role of cell type-specific gene regulation in Parkinson’s disease heritability. NPJ Park. Dis., 5, 6. 10.1038/s41531-019-0076-6 PMC6470136 31016231

[btab175-B37] Sawada H. et al (2013) Catecholamines and neurodegeneration in Parkinson’s Disease—from diagnostic marker to aggregations of α-synuclein. Diagnostics, 3, 210–221. 26835675 10.3390/diagnostics3020210 PMC4665535

[btab175-B38] Skene N.G. , Grant S.G.N. (2016) Identification of vulnerable cell types in major brain disorders using single cell transcriptomes and expression weighted cell type enrichment. Front. Neurosci., 10, 16. 26858593 10.3389/fnins.2016.00016 PMC4730103

[btab175-B39] The GTEx Consortium. et al (2015) The Genotype-Tissue Expression (GTEx) pilot analysis: multitissue gene regulation in humans. Science, 348, 648–660. 25954001 10.1126/science.1262110 PMC4547484

[btab175-B40] Truong P.H. et al (2019) Amyloid precursor protein and amyloid precursor-like protein 2 have distinct roles in modulating myelination, demyelination, and remyelination of axons. Glia, 67, 525–538. 30506868 10.1002/glia.23561

[btab175-B41] Tsoucas D. et al (2019) Accurate estimation of cell-type composition from gene expression data. Nat. Commun., 10, 2975. 31278265 10.1038/s41467-019-10802-z PMC6611906

[btab175-B42] van Dam S. et al (2017) Gene co-expression analysis for functional classification and gene–disease predictions. Brief. Bioinform., 19, 575–592. 10.1093/bib/bbw139 PMC6054162 28077403

[btab175-B43] Wakabayashi K. et al (2000) NACP/α-synuclein-positive filamentous inclusions in astrocytes and oligodendrocytes of Parkinson’s disease brains. Acta Neuropathol. (Berl), 99, 14–20. 10651022 10.1007/pl00007400

[btab175-B44] Wang X. et al (2019) Bulk tissue cell type deconvolution with multi-subject single-cell expression reference. Nat. Commun, 10, 380. 30670690 10.1038/s41467-018-08023-x PMC6342984

[btab175-B45] Wolfe C.J. et al (2005) Systematic survey reveals general applicability of “guilt-by-association” within gene coexpression networks. BMC Bioinformatics, 6, 227. 16162296 10.1186/1471-2105-6-227 PMC1239911

[btab175-B46] Zarranz J.J. et al (2004) The new mutation, E46K, of α-synuclein causes parkinson and Lewy body dementia: new α-synuclein gene mutation. Ann. Neurol., 55, 164–173. 14755719 10.1002/ana.10795

[btab175-B47] Zeisel A. et al (2015) Cell types in the mouse cortex and hippocampus revealed by single-cell RNA-seq. Science, 347, 1138–1142. 25700174 10.1126/science.aaa1934

[btab175-B48] Zeisel A. et al (2018) Molecular architecture of the mouse nervous system. Cell, 174, 999–1014.e22. 30096314 10.1016/j.cell.2018.06.021 PMC6086934

[btab175-B49] Zhang Y. et al (2016) Purification and characterization of progenitor and mature human astrocytes reveals transcriptional and functional differences with mouse. Neuron, 89, 37–53. 26687838 10.1016/j.neuron.2015.11.013 PMC4707064

[btab175-B50] Zhu L. et al (2018) A unified statistical framework for single cell and bulk RNA sequencing data. Ann. Appl. Stat., 12, 609–632. 30174778 10.1214/17-AOAS1110 PMC6114100

