# Supplementary material for: Modeling multifunctionality of genes with secondary gene co-expression networks in human brain provides novel disease insights
Source: Bioinformatics. 2021 Mar 18;37(18):2905–11. doi: 10.1093/bioinformatics/btab175 (PMC8479669; doi:10.1093/bioinformatics/btab175)
Supplement: btab175_Supplementary_Data [file btab175_supplementary_data.zip › suppMethodsFinal.docx]

# Supplementary results

# Introduction

The main idea under GSMCA is to better identify the number of functions and cell types ascribed to a gene in bulk-tissue co-expression networks. In that way, we are able to observe that multifunctional genes will tend to generate more GMSCA predictions and, on the other hand, highly specialised genes will tend to appear as functional only on a specific cell type. To further demonstrate the biological significance of GMSCA’s predictions, we have considered known multifunctional genes in the brain as positive controls and genes known to have very specific function in certain brain cell types as negative controls. For the positive controls, we manually curated genes involved in general metabolic pathways in the brain such as, relaxin signalling pathway, calcium signalling pathway and arginine biosynthesis pathway. For example, it is the case of nitric oxide synthase (NOS) (Forstermann and Sessa, 2012) that it is involved in these pathways in different cell types. For the negative control, we have used a list of genes with specific functions and highly expressed in Purkinje cells in the cerebellum.

The respective sizes for the calcium signalling, the relaxin pathway, the arginine biosynthesis pathway and the negative controls are 240, 129, 21 and 12 genes. For any of the tissues we consider in this paper, and any of the positive control gene sets, GMSCA should generate more multifunctional predictions for those genes than for any random gene set of the same size. On the other hand, for the negative controls GMSCA should generate similar if not lower multifunctional predictions than random genes of the same size. To verify this expectation, we focused our analyses on the 13 GTEx tissues. We obtained the average number of different GMSCA multifunctionality predictions per gene averaged across all 13 tissues, for the three positive and the negative controls. To appropriately calibrate those estimates with respect to what we would get with a same-size randomly chosen gene sets, we obtained the average of multifunctional predictions for 1000 same-size gene. And then we got a ratio of the estimates for the four datasets, and their respective average of 1000 random same-size gene sets. We observe the ratios 1.28, 1.42 and 1.3 for the arginine, calcium signalling and relaxin pathways respectively, and a ratio of 1.01 for the negative controls. This suggests GMSCA gathers multifunctional information in an appropriate manner.

# Supplementary methods

# Hard versus soft clustering approaches

GMSCA rests on the assumption that the clustering algorithm behind the approach produces a disjunct partition of genes, i.e., all genes belong to a cluster, and only one. The techniques generating such type of partitions are referred to as hard clustering techniques. Because of this, a gene cluster predominantly enriched for markers of a specific cell type implies that all genes within the cluster are said to be functional for that cell type.

As an alternative to hard clustering, we also have soft clustering. In fact, there is a variety of clustering approaches that create overlapping partitions such that the points in the clustered space may belong to more than one cluster through a membership function. In the context of gene clustering, such membership function is m(g,c) where g is a gene and c is a cluster of the overlapping partition, which generates values in [0,1], and higher values of m(g,c) somewhat indicate higher confidence in assigning g to c.

Examples of soft clustering approaches include fuzzy clustering (D’Urso and Gil, 2017) in which all genes belong to all clusters with a certain degree and probabilistic clustering, which assign a probability distribution for each gene of belonging to all clusters. A not-so-soft alternative to hard clustering is used in community detection algorithms (Ahn et al., 2010; Ding et al., 2016; Palla et al., 2005). These algorithms acknowledge and detect partial node and link overlap between communities or clusters.

We recognize it is useful to use clustering models that are capable of detecting and managing multiple memberships of nodes to different clusters or modules. This is especially useful when the frontiers between clusters are blurred. Examples of this include clustering of press news documents depending on the topics they address (e.g., politics, sports, economics, health, etc.) and also proteins and genes acting in different pathways.

GMSCA uses a hard clustering approach for a number of reasons. We understand gene functionality across different cell types as hard categories. In the sense that a gene is said to be functional for a cell type or not.

On the opposite case, the mapping between genes and their possible cell type functional categories could be approached in two different manners, and none of them would be satisfactory. On the one hand, we could assign a cell type functional category to a gene when m(g,c) is greater than a certain threshold but there is the problem on how to decide on a suitable threshold. On the other hand, we could just say that a gene g is assigned to all cell type categories with a certain degree in [0,1]. However, we are not sure of what would be the best way to manage such predictions because interpreting a probability distribution for each gene belonging to all cell categories would be far from being trivial. In consequence, GMSCA maps genes to a subset of all cell type function categories. Consequently, if GMSCA predicts that g is functional in a cell type, it is because there is strong evidence for that, as that gene was found in a primary or secondary network module enriched for cell markers of that cell type. If GMSCA does not detect that g is functional on another cell type, this just means we found no evidence for that. This way of using GCNs and GMSCA predictions makes interpretation of results very easy.

# Extended explanation of the GBA paradigm and its use within GMSCA

A previous step before GMSCA generates predictions is to create a clustering of genes. Then, those gene clusters are annotated (1) for function as a group (i.e., by looking for annotation terms in biological databases which are enriched for genes belonging to those modules) and (2) for cell type (i.e., by looking at the statistically significant overlap with cell marker genes). Both groups of annotation compound the basis for the triplets <gene, cell type, function> that GMSCA generates.

The cell functionality annotation of a cluster/module of genes G={g_1_, g_2_, …, g_n_} consists on assigning a cell type to all genes in G. And then, give that MSCA predicts all genes in G to be functional to a cell type, it proceeds to the functional annotation of G, which assigns a set of functional terms T={t_1_, t_2_, …, t_m_} to the genes in G. In consequence, GMSCA generates n new triplets <g, c, T>, where g is a gene in G, c is the functional cell type of G and T is the set of functional terms assigned to G. Note that, what GMSCA implicitly assumes when it does that is the Guilty By Association principle (Gillis and Pavlidis, 2011; Wolfe et al., 2005). Because it assigns all terms in T to all single genes in G and predicts all genes in G are functional in cell type c even when not all genes in G may have been participating in the generation of all terms in T or the assignment of cell type c.

Therefore, when other clustering methods just predict the clustering structure itself, GMSCA predictions are a by-product of such clustering structure, i.e., it requires that clustering to generate triplets. In consequence, GMSCA does not depend on a specific clustering algorithm to generate the triplets. In fact, it may well use any other hard clustering approach. Its current implementation is based on the CoExpNets R package (Botía, Juan A. et al., 2017) for the construction of GCNs.

The explanation about how GMSCA generates a set of functional annotation terms T for all modules in either a PGCN (primary) or SGCN (secondary) follows. Gene set annotation is a classical task in bioinformatics which entails a statistical procedure that assigns descriptive terms to genes in an attempt to describe their function (Carbon et al., 2009; Conesa et al., 2005; Liberzon et al., 2015; Supek et al., 2011). In order to do that, we need a statistical approach to find significant links between genes and annotation terms, besides a database of terms and their association to genes. These databases may come in two alternative forms, one as an ontology and the other one as a pathway.

The current standard ontology model for biological annotation is the Gene Ontology (GO) (The Gene Ontology Consortium, 2017). This is a formal model of concepts structure which describes biological processes, molecular functions and cellular components which emerge from what is currently known about biology. Such structure comes in the form of a graph and all concepts (i.e., the terms as we call them) have genes associated with them. For example, in the following link takes us to all the ontology terms linked to the CSF1R gene at GO:

<http://amigo.geneontology.org/amigo/gene_product/UniProtKB:P07333>

All these terms will normally have many genes linked to it. Therefore, the statistical procedure that detects terms enriched for genes in the modules of PGCNs and SGCNs works by finding statistically significant overlaps between genes in network modules and genes linked to GO terms. Thus, when a module is associated to a term in the GO ontology this means that the module is enriched for genes associated to the term at GO. GMSCA performs this analysis at all modules in both PGCN and SGNC networks. Therefore, many terms at T come from this analysis and they end up as the third element of the triplets <gene, cell type, function>.

When it comes down to pathways, the most used pathway databases are REACTOME (Fabregat et al., 2018) and KEGG (Kanehisa et al., 2016). A biological pathway is a set of events between chemicals (i.e., gene products) leading to a change in the cell. As a way of example, we can inspect the same gene, CSF1R at the REACTOME database through this link:

<https://reactome.org/content/detail/R-HSA-197667>

At that page, we can use the links under “Locations in the PathwayBrowser” to see all pathways associated with this gene (More information about REACTOME: <https://reactome.org/what-is-reactome>). When we use either REACTOME or KEGG to annotate GNC modules, the terms are, this time, pathway names and the statistical procedures to link terms to GCN modules is basically the same, i.e., detecting significant overlap between genes in modules and genes linked to pathways. We use gProfileR to automatize functional enrichment tests with GO, REACTOME and KEGG within the CoExpNets R package.

# Potential sources of variability affecting GMSCA results

GMSCA predictions depend on GCNs and GCN models are based on expression profiling. Therefore, the same sources of variability (either technical or biological) affecting the level of technical noise and quality of expression profiling will have an effect on GCNs and, therefore, on GMSCA predictions. Amongst the technical sources of variability, we can include the technology used to obtain the expression profiling (i.e., microarrays, RNA-seq, and the differences in approach between commercial products), the pipeline used to deal with the expression profiling to obtain a GCN (we outline our basic pipeline at the main section of this paper), the number of samples available to create the network and the cell composition heterogeneity across samples in bulk tissue. Note all networks used in this paper are derived from bulk tissue.

# 2.3.1 Influence of sample availability on GMSCA

In order to illustrate how sample availability influences the features of a GCN created with GMSCA, we performed some experiments as follows. First, we performed a small analysis, with all the frontal cortex sample sets available, FCTX from 10UKBEC, FCortex from GTEx and the NotAd samples from ROSMAP. We wanted to investigate whether the number of samples used to create a GCN has influence in the final number of modules of the GCN. For such purpose, and within each tissue sample set, we start by randomly choosing 40 samples to create a network with them. In the following steps, we successively create new networks by increasing the number of samples by 10 each time, until we reach the available number of samples. Then, we report the number of modules for each network. To cope with randomness, this whole process is repeated seven times. For example, the FCTX sample set has 83 samples, within each repetition of the process, we create a GCN of 40 samples, we add 10 more samples to create one of 50, 60, 70 and 80 samples. At the end, only for FCTX we create 35 GCNs and measure their modules. Both FCortex and NotAD sample sets are treated in the same way.

The results are depicted in figure A below. The left plot corresponds to FCTX at 10UKBEC and the left-most whisker-plot corresponds to the seven GCNs created with 40 random samples. In that plot, we note a decrease in the number of modules, on average, as the sample set grows, and the tendency suggests that the most plausible number of modules, at least for this sample set, would be around 25. This number may be even less for FCortex, around 20, with 108 samples. But clearly, this tendency for the number of modules to decrease when more samples are used holds for the three sample sets. We conclude that these experiments suggest that for most datasets, sample availability influence the number of modules the GCN will present. A GCN with more modules, and hence smaller in genes, lead to more chances for the detection of genuine enrichment of gene markers but less net number of GMSCA triplets (as many as genes contained at the module).

# 2.3.2 Influence of gene marker sets on GMSCA results

The gene marker sets used by the current version of GMSCA implementation are reused from the CoExpNets package. All gene marker sets are accessible at CoExpNets´ GitHub:

<https://github.com/juanbot/CoExpNets/tree/master/inst/ctall>

Each text file under this folder represents a different cell marker set. All files include, as the first line, a tag with the specific cell type they are tagging, followed by the gene markers, 1 gene per line. CoExpNets´ gene marker sets include human brain cell type markers identified at the Darmanis paper from 2015 (Darmanis et al., 2015), the Neuroexpresso project (Mancarci et al., 2017) and the markers from the brain list of WGCNA (Miller et al., 2011). Note that in the current version of GMSCA, we only consider four main types of cells, i.e., neurons, microglia, astrocytes and oligodendrocytes. GMSCA treats all files of any subtype as if they were of the general type. In consequence, when GMSCA tests any GCN module for enrichment of a given cell type, it tests that module against all gene sets from CoExpNets, under that main cell type, i.e., it generates multiple tests. This notably increases the possibility of detecting biologically genuine enrichments. In that way, we wanted to investigate whether the variability in the size of the gene markers has some effect on GMSCA predictions. Therefore, we aimed to look for a possible association between the gene marker sets sizes and the number of positive enrichments detected. CoExpNets gene marker sets have an average size of 140 genes (see Figure 1d).

We restrict the analysis here to the 13 GTEx networks. We created regression models for all GTEx PGCNs, y ~ x, where x is the gene marker set size and y is the -log10(pval) of the Fisher´s Exact test on the overlap between module genes and marker genes. We only consider significant test results (i.e. P < 0.05). We actually find a significant association of those variables (i.e., Pearson correlation of gene set size and -log10() of the test p-value is 0.64) suggesting there is an inflation of the significance tests. The linear regression models appear depicted at the plots in figure B below (we just include plots for Substantia Nigra and Putamen but the tendency is the same for 13 networks). The raw points appear in red and the regression model is the red line for those points.

To minimize such bias we designed a correction procedure. We apply to the tests p-values by regressing the gene set size out of the -log10() transformation of the p-values. It works by performing a two steps post-correction phase to correct the p-values computed by the Fisher´s Exact test in charge of detecting enrichment of markers within modules. In a first step, we use a Bonferroni correction that accounts for multiple testing, i.e., it considers the number of modules and marker sets involved in the tests. In a second step, GMSCA creates a linear model of the relation between p-values in overlap tests and marker sets size. This linear model is finally used to predict the mean p-value obtained by each marker set and, these values are then subtracted from the actual p-values found for each marker set in each module of the network. The results appear at the same plots, now in blue color. By applying this two-step procedure, the number of significant tests decreases to 40% of the original significant tests.

References

Ahn, Y.-Y., Bagrow, J.P., and Lehmann, S. (2010). Link communities reveal multiscale complexity in networks. Nature *466*, 761–764.

Botía, Juan A., Vandrovcova, J., Forabosco, P., Guelfi, S., D’Sa, K., Hardy, J., Lewis, C.M., Ryten, M., and Weale, M.E. (2017). An additional k-means clustering step improves the biological features of WGCNA gene co-expression networks. BMC Syst. Biol. *11*.

Carbon, S., Ireland, A., Mungall, C.J., Shu, S., Marshall, B., Lewis, S., the AmiGO Hub, and the Web Presence Working Group (2009). AmiGO: online access to ontology and annotation data. Bioinformatics *25*, 288–289.

Conesa, A., Gotz, S., Garcia-Gomez, J.M., Terol, J., Talon, M., and Robles, M. (2005). Blast2GO: a universal tool for annotation, visualization and analysis in functional genomics research. Bioinformatics *21*, 3674–3676.

Darmanis, S., Sloan, S.A., Zhang, Y., Enge, M., Caneda, C., Shuer, L.M., Hayden Gephart, M.G., Barres, B.A., and Quake, S.R. (2015). A survey of human brain transcriptome diversity at the single cell level. Proc. Natl. Acad. Sci. *112*, 7285–7290.

Ding, Z., Zhang, X., Sun, D., and Luo, B. (2016). Overlapping Community Detection based on Network Decomposition. Sci. Rep. *6*.

D’Urso, P., and Gil, M.Á. (2017). Fuzzy data analysis and classification: Special issue in memoriam of Professor Lotfi A. Zadeh, father of fuzzy logic. Adv. Data Anal. Classif. *11*, 645–657.

Fabregat, A., Jupe, S., Matthews, L., Sidiropoulos, K., Gillespie, M., Garapati, P., Haw, R., Jassal, B., Korninger, F., May, B., et al. (2018). The Reactome Pathway Knowledgebase. Nucleic Acids Res. *46*, D649–D655.

Forstermann, U., and Sessa, W.C. (2012). Nitric oxide synthases: regulation and function. Eur. Heart J. *33*, 829–837.

The Gene Ontology Consortium (2017). Expansion of the Gene Ontology knowledgebase and resources. Nucleic Acids Res. *45*, D331–D338.

Gillis, J., and Pavlidis, P. (2011). The Impact of Multifunctional Genes on “Guilt by Association” Analysis. PLoS ONE *6*, e17258.

Kanehisa, M., Sato, Y., Kawashima, M., Furumichi, M., and Tanabe, M. (2016). KEGG as a reference resource for gene and protein annotation. Nucleic Acids Res. *44*, D457–D462.

Liberzon, A., Birger, C., Thorvaldsdóttir, H., Ghandi, M., Mesirov, J.P., and Tamayo, P. (2015). The Molecular Signatures Database Hallmark Gene Set Collection. Cell Syst. *1*, 417–425.

Mancarci, B.O., Toker, L., Tripathy, S.J., Li, B., Rocco, B., Sibille, E., and Pavlidis, P. (2017). Cross-Laboratory Analysis of Brain Cell Type Transcriptomes with Applications to Interpretation of Bulk Tissue Data. Eneuro *4*, ENEURO.0212-17.2017.

Miller, J.A., Cai, C., Langfelder, P., Geschwind, D.H., Kurian, S.M., Salomon, D.R., and Horvath, S. (2011). Strategies for aggregating gene expression data: The collapseRows R function. BMC Bioinformatics *12*, 322.

Palla, G., Derényi, I., Farkas, I., and Vicsek, T. (2005). Uncovering the overlapping community structure of complex networks in nature and society. Nature *435*, 814–818.

Supek, F., Bošnjak, M., Škunca, N., and Šmuc, T. (2011). REVIGO Summarizes and Visualizes Long Lists of Gene Ontology Terms. PLoS ONE *6*, e21800.

Wolfe, C.J., Kohane, I.S., and Butte, A.J. (2005). Systematic survey reveals general applicability of “guilt-by-association” within gene coexpression networks. BMC Bioinformatics *6*, 227.
