# Supplementary figures and images for: Modeling multifunctionality of genes with secondary gene co-expression networks in human brain provides novel disease insights
Source: Bioinformatics. 2021 Mar 18;37(18):2905–11. doi: 10.1093/bioinformatics/btab175 (PMC8479669; doi:10.1093/bioinformatics/btab175)

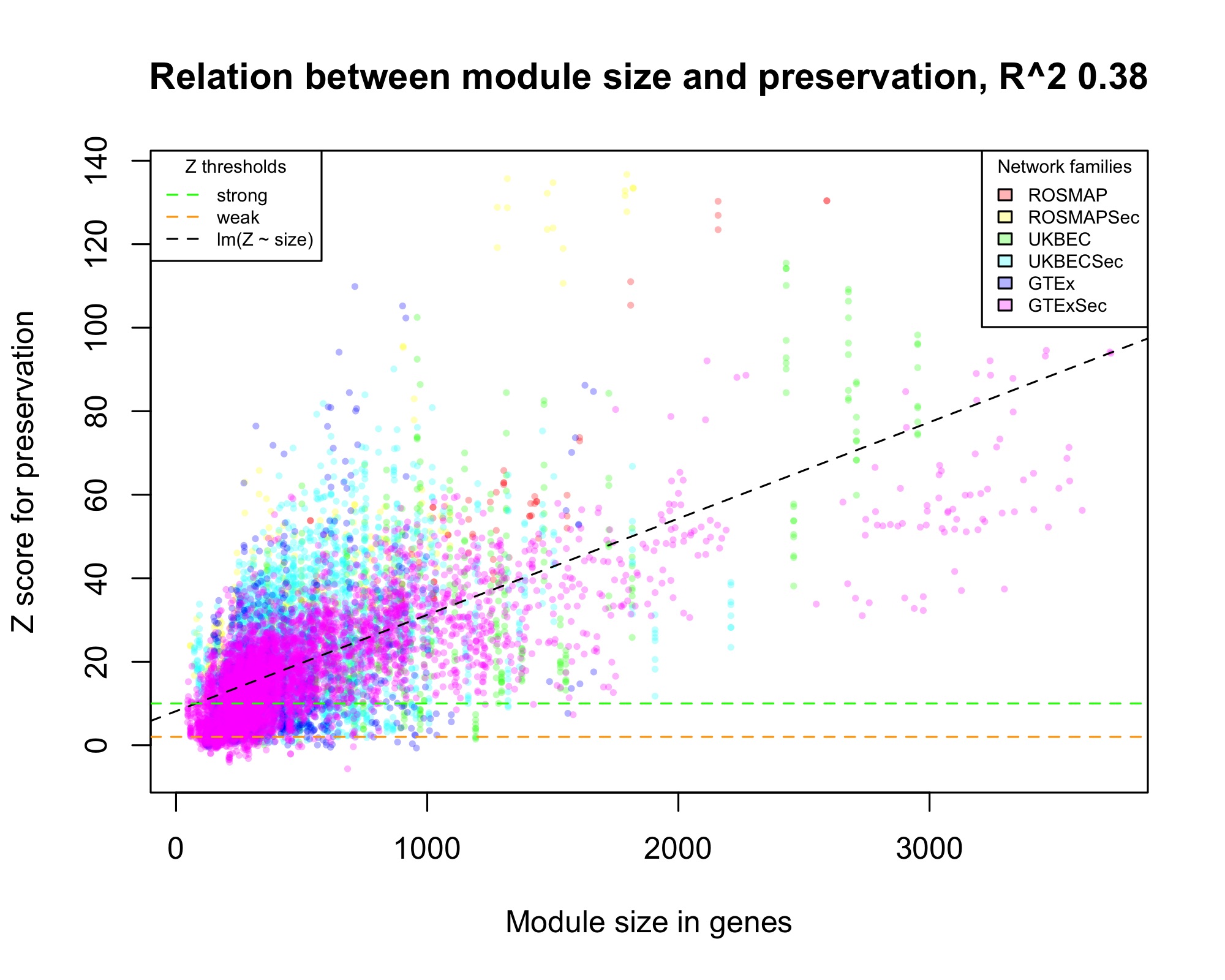

Supplement: btab175_Supplementary_Data [file btab175_supplementary_data.zip › SuppFig1.jpg]

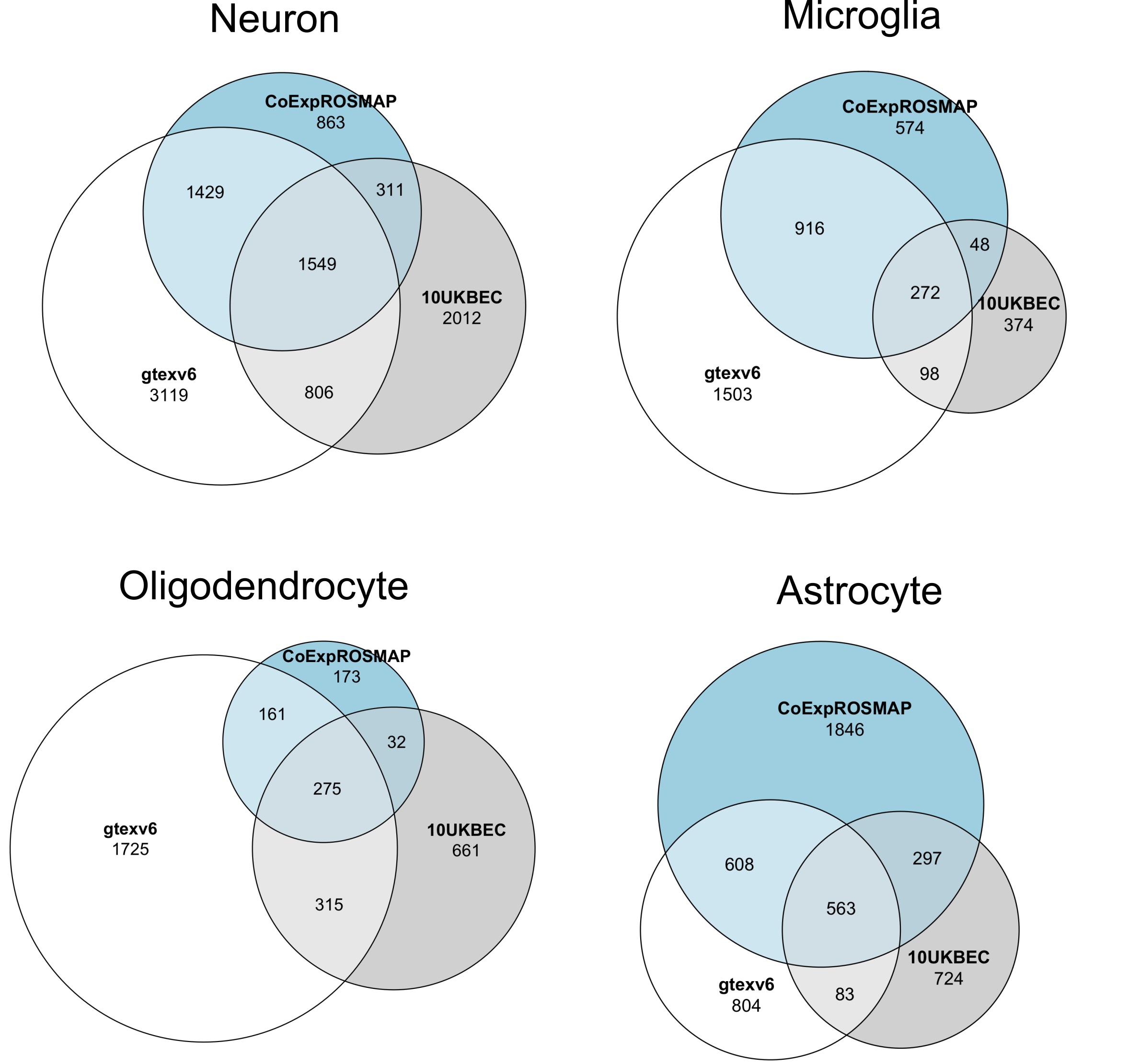

Supplement: btab175_Supplementary_Data [file btab175_supplementary_data.zip › SuppFig2.jpg]

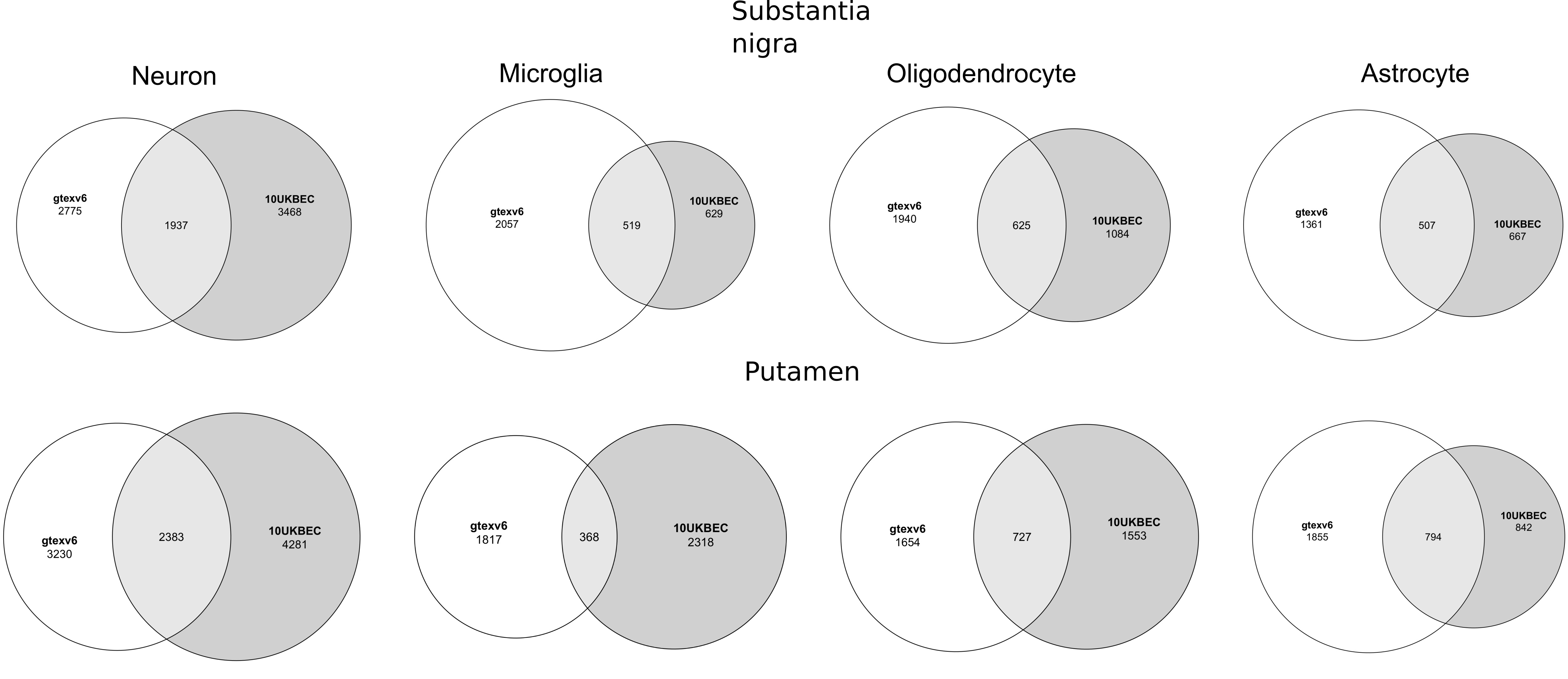

Supplement: btab175_Supplementary_Data [file btab175_supplementary_data.zip › SuppFig3.jpg]

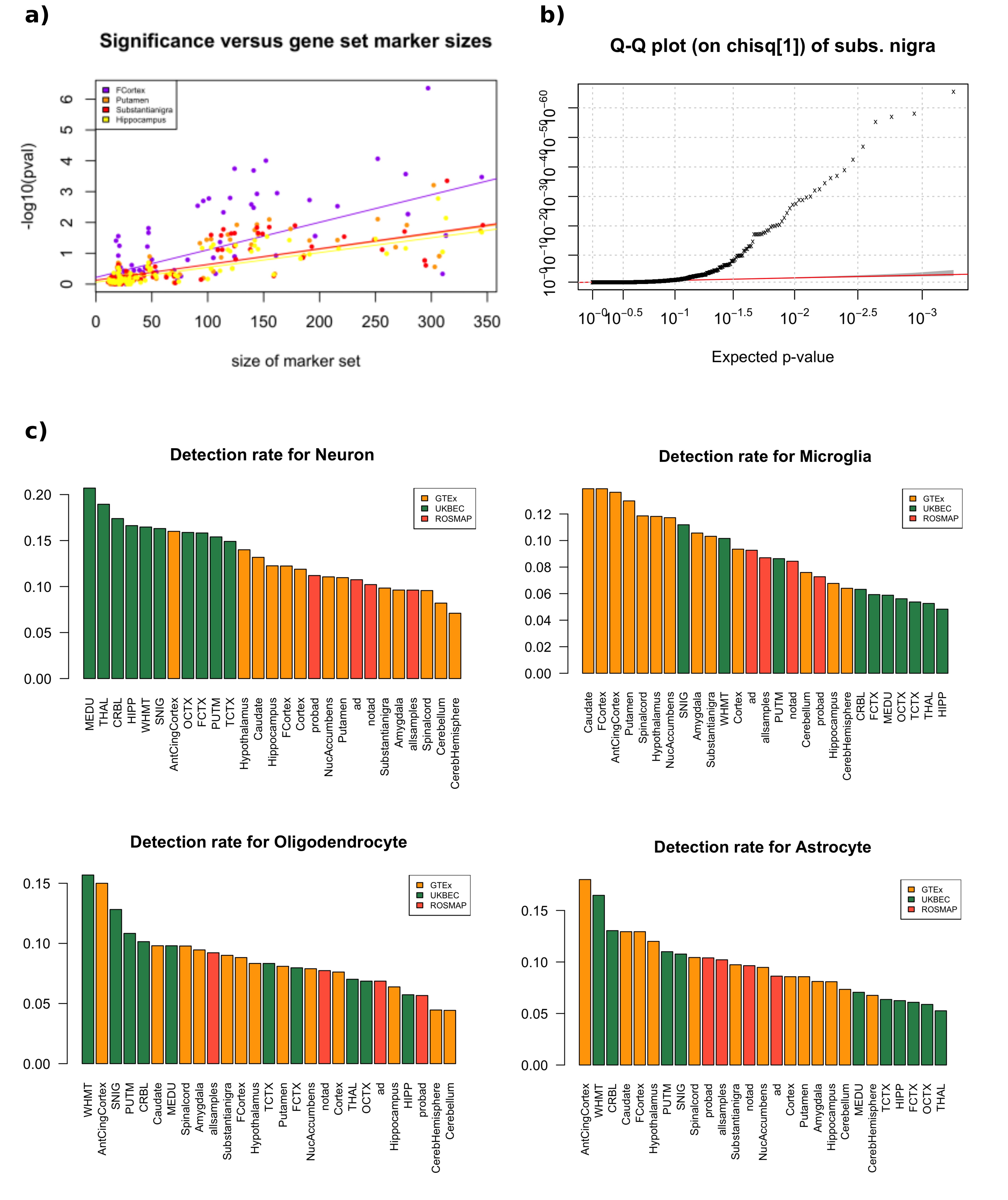

Supplement: btab175_Supplementary_Data [file btab175_supplementary_data.zip › SuppFig4.jpg]
